# Evaluation of the VITEK 2 AST-N439 card for susceptibility testing of novel β-lactam/β-lactamase inhibitor combinations and colistin in carbapenem-non-susceptible gram-negative bacilli

**DOI:** 10.1128/spectrum.00166-25

**Published:** 2025-08-21

**Authors:** Tae Yeul Kim, Jin Yang Baek, Eunsang Suh, Jun-Ki Lee, Hui-Jin Yu, Jae-Hoon Ko, Hee Jae Huh, Nam Yong Lee

**Affiliations:** 1 Department of Laboratory Medicine and Genetics, Samsung Medical Center, Sungkyunkwan University School of Medicine, Seoul, Republic of Korea; 2 Asia Pacific Foundation for Infectious Diseases (APFID), Seoul, Republic of Korea; 3 Division of Infectious Diseases, Department of Medicine, Samsung Medical Center, Sungkyunkwan University School of Medicine, Seoul, Republic of Korea; 4 Department of Laboratory Medicine, Seoul Medical Center, Seoul, Republic of Korea; 5 Department of Medical Device Management and Research, Samsung Advanced Institute for Health Sciences and Technology, Sungkyunkwan University, Seoul, Republic of Korea; The George Washington University School of Medicine and Health Sciences, Washington, DC, USA

**Keywords:** susceptibility testing, VITEK 2, novel BL/BLI combinations, colistin, gram-negative bacilli

## Abstract

We assessed the performance of the VITEK 2 AST-N439 card for susceptibility testing of novel β-lactam/β-lactamase inhibitor (BL/BLI) combinations and colistin in carbapenem-non-susceptible gram-negative isolates. A total of 425 clinical isolates, including *Enterobacterales* (*n* = 242), *Pseudomonas aeruginosa* (*n* = 97), and *Acinetobacter baumannii* (*n* = 86), were tested using VITEK 2, with broth microdilution as the reference standard. Minimum inhibitory concentrations were interpreted according to Clinical and Laboratory Standards Institute breakpoints. The essential agreement (EA), categorical agreement (CA), very major error rate, and major error rate for VITEK 2 were 90.3%, 97.6%, 1.8%, and 2.5% for ceftazidime/avibactam; 81.5%, 89.0%, 7.0%, and 4.7% for ceftolozane/tazobactam; 96.5%, 90.9%, 3.4%, and 1.2% for imipenem/relebactam; and 94.6%, 93.0%, 15.8%, and 3.2% for meropenem/vaborbactam. For colistin, VITEK 2 demonstrated an EA of 89.2% and a CA of 85.4%. Our findings suggest that VITEK 2 has suboptimal performance for novel BL/BLI combinations in carbapenem-non-susceptible gram-negative isolates. Furthermore, despite changes to the colistin formulation used in the VITEK 2 card, it remains an unreliable method for detecting colistin resistance.

**IMPORTANCE:**

Rapid and accurate antimicrobial susceptibility testing (AST) is essential for managing infections caused by multidrug-resistant gram-negative bacilli. This study evaluated the performance of the VITEK 2 AST-N439 card for susceptibility testing of novel BL/BLI combinations and colistin in carbapenem-non-susceptible gram-negative isolates. Our findings revealed significant limitations, including suboptimal performance for novel BL/BLI combinations and unreliable detection of colistin resistance, even with the updated formulation. These results underscore the need for cautious interpretation of VITEK 2 results and highlight the importance of optimizing its performance to enhance antibiotic decision-making.

## INTRODUCTION

Infections caused by multidrug-resistant (MDR) gram-negative bacteria, including carbapenem-resistant *Enterobacterales* (CRE), carbapenem-resistant *Pseudomonas aeruginosa* (CRPA), and carbapenem-resistant *Acinetobacter baumannii*, continue to pose a major challenge to global public health (1, 2). Older antibiotics, such as colistin, fosfomycin, and tigecycline, used to treat these infections, are constrained by limited efficacy, safety concerns (e.g., nephrotoxicity associated with colistin), and the rise of resistance (3). To address these limitations, the development and introduction of newer antibiotics effective against MDR gram-negative bacteria are urgently needed (4). Since the mid-2010s, several novel β-lactam/β-lactamase inhibitor (BL/BLI) combinations, including ceftazidime/avibactam, ceftolozane/tazobactam, imipenem/relebactam, and meropenem/vaborbactam, have been approved for treating infections caused by MDR gram-negative bacteria and are now widely utilized in clinical practice (3, 5). However, resistance to these agents is increasingly reported among CRE and CRPA isolates (6–10), underscoring the urgent need for accurate and widely accessible antimicrobial susceptibility testing (AST) methods to effectively guide antibiotic therapy.

In March 2024, the VITEK 2 AST-N439 card (bioMérieux, Marcy l'Etoile, France) was approved in Korea for susceptibility testing of MDR gram-negative bacilli and is now widely used in clinical microbiology laboratories. This card includes novel BL/BLI combinations: ceftazidime/avibactam, ceftolozane/tazobactam, imipenem/relebactam, and meropenem/vaborbactam. Although the performance of VITEK 2 for these agents has been assessed in several studies (11–16), it has not yet been evaluated in Korea. Additionally, this card includes a new formulation of colistin (cs02n), the performance of which is not yet well established. The aim of this study was to evaluate the performance of the VITEK 2 AST-N439 card for susceptibility testing of novel BL/BLI combinations and colistin in carbapenem-non-susceptible gram-negative isolates, for which this card is primarily intended.

## MATERIALS AND METHODS

### Bacterial strains

This study included 425 non-duplicate gram-negative isolates classified as carbapenem-non-susceptible by routine AST with the VITEK 2 system between January 2019 and June 2024. Carbapenem non-susceptibility was defined as intermediate or resistant to at least one of the carbapenems tested by VITEK 2: ertapenem and imipenem for *Enterobacterales*, and imipenem and meropenem for *P. aeruginosa* and *A. baumannii*. The collection consisted of 242 *Enterobacterales*, 97 *P*. *aeruginosa*, and 86 *A*. *baumannii* isolates. *Enterobacterales* species included *Klebsiella pneumoniae* (*n* = 78), *Escherichia coli* (*n* = 54), *Enterobacter cloacae* complex (*n* = 38), *Klebsiella aerogenes* (*n* = 23), *Citrobacter freundii* complex (*n* = 22), *Serratia marcescens* (*n* = 13), *Klebsiella oxytoca* (*n* = 8), *Citrobacter koseri* (*n* = 3), and *Proteus mirabilis* (*n* = 3). Isolates were obtained from various sources, with the majority isolated from blood cultures (*n* = 259, 60.9%), followed by urine (*n* = 54, 12.7%), peritoneal fluid (*n* = 32, 7.5%), respiratory specimens (*n* = 28, 6.6%), bile (*n* = 16, 3.8%), pus (*n* = 10, 2.4%), and stool (*n* = 8, 1.9%). The remaining 18 isolates (4.2%) were recovered from other specimen types. The distribution of carbapenemase types across all isolates is presented in Table S1.

Frozen stock isolates were subcultured twice on Columbia agar with 5% sheep blood prior to testing with the VITEK 2 system and broth microdilution (BMD). Before susceptibility testing, the species of these isolates were confirmed by matrix-assisted laser desorption/ionization time-of-flight mass spectrometry (VITEK MS, bioMérieux). VITEK 2 and BMD testing were performed by two experienced technicians. For procedural convenience, two bacterial plates were prepared for each isolate, with one plate assigned to each technician. Each technician used their assigned plate to prepare the inoculum for the corresponding method.

### Antimicrobial susceptibility testing

AST was conducted using the VITEK 2 system (software version 9.04.4) with the AST-N439 card, following the manufacturer’s instructions. BMD was used as the reference method and performed according to the Clinical and Laboratory Standards Institute (CLSI) M07-A10 guidelines (17). Briefly, bacterial suspensions were prepared in cation-adjusted Mueller-Hinton broth to achieve a final concentration of 5 × 10^5^ CFU/mL and added to untreated polystyrene 96-well microplates containing serial twofold dilutions of antibiotics. The plates were incubated for 16–20 hours at 35°C, and minimum inhibitory concentrations (MICs) were determined by visual inspection. *E. coli* American Type Culture Collection (ATCC) 25922 and *P. aeruginosa* ATCC 27853 were used as quality control strains. MICs obtained from VITEK 2 and BMD were interpreted using breakpoints outlined in CLSI Supplement M100, 34th edition (18).

### Data analysis

VITEK 2 results were compared with those obtained from BMD, which was used as the reference method. Discrepant results between the two methods were categorized as very major errors (VMEs), where isolates were classified as susceptible by VITEK 2 but resistant by BMD; major errors (MEs), where isolates were classified as resistant by VITEK 2 but susceptible by BMD; and minor errors (mEs), where isolates were intermediate by one method but either susceptible or resistant by the other. Categorical agreement (CA) was defined as the percentage of VITEK 2 results matching the susceptibility categories determined by BMD. Essential agreement (EA) was calculated as the percentage of VITEK 2 MIC results falling within one twofold dilution of BMD MIC results. Discrepancy resolution testing for VMEs and MEs was conducted using one-time triplicate BMD testing with separate bacterial suspensions, in accordance with International Organization for Standardization (ISO) 20776-2:2007 standards (19). Performance characteristics were determined after resolving the discrepancies. Acceptable performance was assessed based on ISO criteria: CA ≥90%, EA ≥90%, ME ≤3%, and VME ≤3% (19).

## RESULTS

### Performance of VITEK 2 for novel BL/BLI combinations

**Fig 1:**
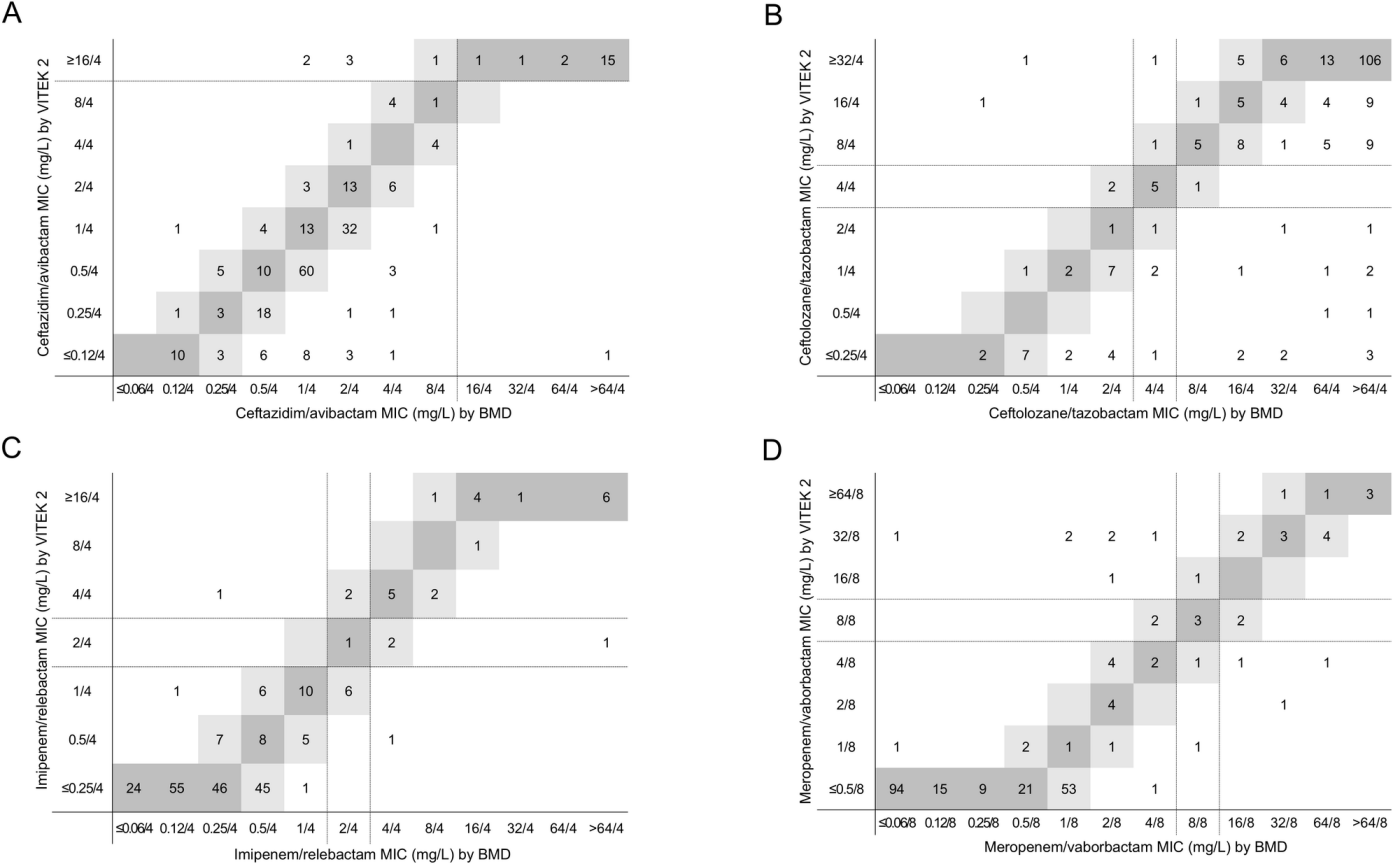
Comparison of MICs for novel BL/BLI combinations between VITEK 2 and BMD in carbapenem-non-susceptible *Enterobacterales*. MICs of ceftazidime/avibactam (**A**), ceftolozane/tazobactam (**B**), imipenem/relebactam (**C**), and meropenem/vaborbactam (**D**) were compared between the two methods. Dark gray represents identical MICs, while light gray indicates a twofold MIC difference. Dotted lines correspond to CLSI breakpoints for each antimicrobial.

Table 1 presents the performance of VITEK 2 relative to BMD for novel BL/BLI combinations in carbapenem-non-susceptible gram-negative bacilli. Based on BMD results, resistance rates for these agents among *Enterobacterales* were 8.3% for ceftazidime/avibactam, 82.8% for ceftolozane/tazobactam, 9.9% for imipenem/relebactam, and 7.9% for meropenem/vaborbactam. Among *Enterobacterales*, CA met the ISO criterion of ≥90% for all agents except ceftolozane/tazobactam, which had a CA of 89.1%. EA met the ISO criterion of ≥90% for imipenem/relebactam and meropenem/vaborbactam; however, EAs for ceftazidime/avibactam and ceftolozane/tazobactam were 87.2% and 76.9%, respectively, not meeting the criterion (Table 1). VITEK 2 showed a tendency to underestimate MICs for these agents compared to BMD (Fig. 1). VME ranged from 4.2% to 15.8%, failing to meet the ISO criterion of ≤3% for all agents. ME met the ISO criterion of ≤3% for ceftazidime/avibactam and imipenem/relebactam. In contrast, MEs for ceftolozane/tazobactam and meropenem/vaborbactam were 6.7% and 3.2%, respectively, not meeting the criterion. mEs were observed in 9 isolates (3.8%) for ceftolozane/tazobactam, 11 isolates (4.5%) for imipenem/relebactam, and 7 isolates (2.9%) for meropenem/vaborbactam. The majority of these (*n* = 5, 10, and 6, respectively) were within EA, despite categorical discrepancies. Overall, VITEK 2 performance among carbapenem-non-susceptible *Enterobacterales* did not meet ISO acceptance criteria for all agents (Table 1). Detailed species-specific performance data are provided in Table S2.

**TABLE 1:** Performance of VITEK 2 for novel BL/BLI combinations in carbapenem-non-susceptible gram-negative isolates^*b*^

Organism/antimicrobial agent	Total tested	BMD			No. (%) of errors			CA (%)	EA (%)
		S	I	R	VME	ME	mE		
*Enterobacterales* (*n* = 242)									
Ceftazidime/avibactam	242	222	N/A	20	1 (5.0)	6 (2.7)	N/A	97.1	87.2
Ceftolozane/tazobactam	238^*a*^	30	11	197	15 (7.6)	2 (6.7)	9 (3.8)	89.1	76.9
Imipenem/relebactam	242	209	9	24	1 (4.2)	1 (0.5)	11 (4.5)	94.6	97.9
Meropenem/vaborbactam	242	217	6	19	3 (15.8)	7 (3.2)	7 (2.9)	93.0	94.6
*P*. *aeruginosa* (*n* = 97)									
Ceftazidime/avibactam	97	61	N/A	36	0 (0)	1 (1.6)	N/A	99.0	97.9
Ceftolozane/tazobactam	97	55	11	31	1 (3.2)	2 (3.6)	8 (8.2)	88.7	92.8
Imipenem/relebactam	97	50	12	35	1 (2.9)	2 (4.0)	15 (15.5)	81.4	92.8
All species combined (*n* = 339)									
Ceftazidime/avibactam	339	283	N/A	56	1 (1.8)	7 (2.5)	N/A	97.6	90.3
Ceftolozane/tazobactam	335	85	22	228	16 (7.0)	4 (4.7)	17 (5.1)	89.0	81.5
Imipenem/relebactam	339	259	21	59	2 (3.4)	3 (1.2)	26 (7.7)	90.9	96.5
Meropenem/vaborbactam	242	217	6	19	3 (15.8)	7 (3.2)	7 (2.9)	93.0	94.6

^*a*^ Four isolates were excluded from analysis because the VITEK 2 system terminated ceftolozane/tazobactam MIC testing due to insufficient growth in the positive control well.

^*b*^ BMD, broth microdilution; CA, categorical agreement; EA, essential agreement; I, intermediate; ME, major error; mE, minor error; N/A, not applicable; R, resistant; S, susceptible; VME, very major error.

**Fig 2:**
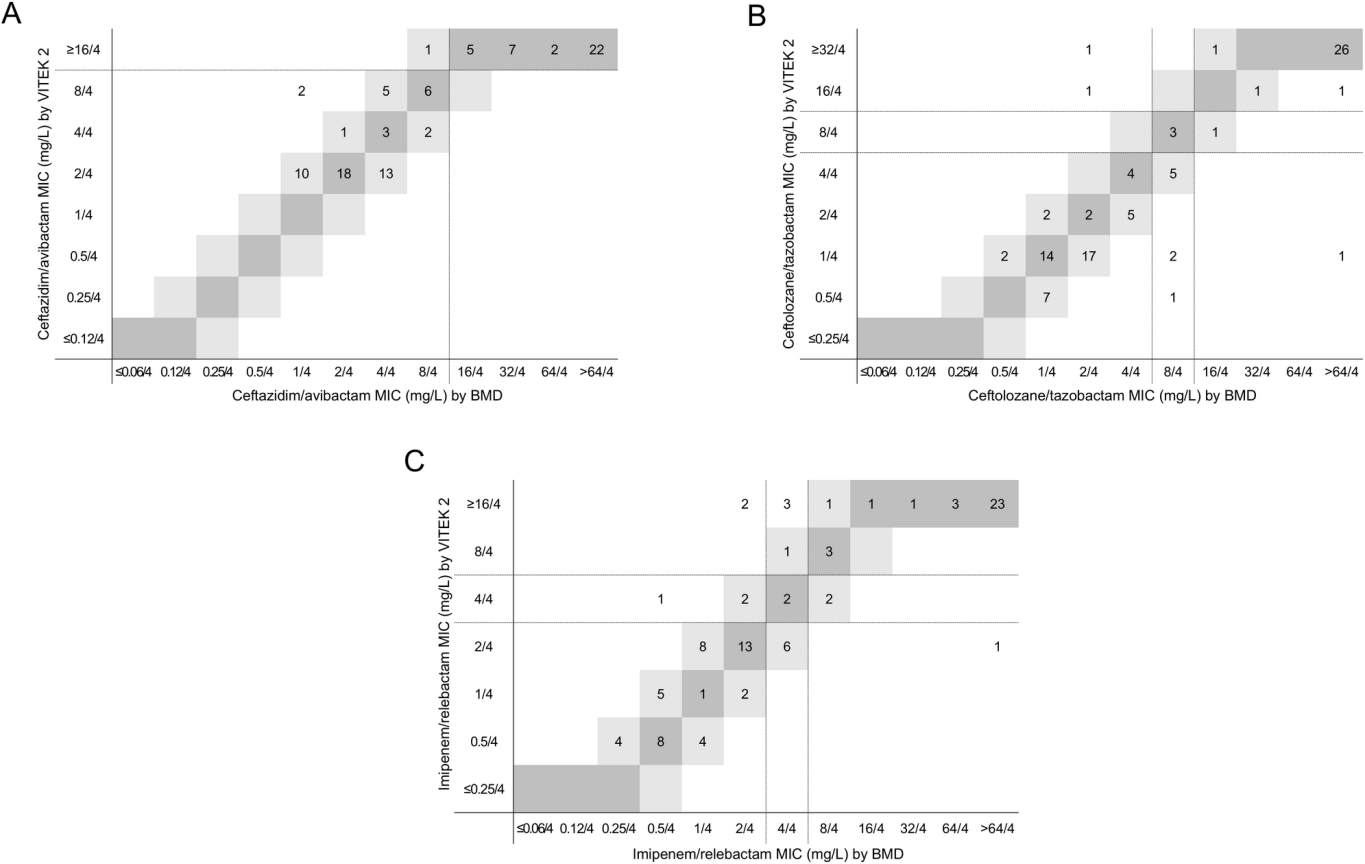
Comparison of MICs for novel BL/BLI combinations between VITEK 2 and BMD in carbapenem-non-susceptible *P. aeruginosa* isolates. MICs of ceftazidime/avibactam (**A**), ceftolozane/tazobactam (**B**), and imipenem/relebactam (**C**) were compared between the two methods. Dark gray represents identical MICs, while light gray indicates a twofold MIC difference. Dotted lines correspond to CLSI breakpoints for each antimicrobial.

For *P. aeruginosa*, resistance rates for novel BL/BLI combinations were 37.1% for ceftazidime/avibactam, 32.0% for ceftolozane/tazobactam, and 36.1% for imipenem/relebactam. CA met the ISO criterion of ≥90% for ceftazidime/avibactam, but CAs for ceftolozane/tazobactam and imipenem/relebactam were 88.7% and 81.4%, respectively, not meeting the criterion (Table 1). The correlation between VITEK 2 and BMD MICs was excellent (Fig. 2), with EA exceeding the ISO criterion of ≥90% for all agents (Table 1). VME met the ISO criterion of ≤3% for ceftazidime/avibactam and imipenem/relebactam, while VME for ceftolozane/tazobactam was 3.2%, not meeting the criterion. ME met the ISO criterion of ≤3% for ceftazidime/avibactam, while MEs for ceftolozane/tazobactam and imipenem/relebactam were 3.6% and 4.0%, respectively, not meeting the criterion. mEs were observed in 8 isolates (8.2%) for ceftolozane/tazobactam and 15 isolates (15.5%) for imipenem/relebactam. Of these, the majority (*n* = 5 and 11, respectively) were within EA, despite categorical discrepancies. Since the CLSI breakpoint for meropenem/vaborbactam in *P. aeruginosa* was unavailable, VITEK 2 performance for this agent was not assessed. Overall, VITEK 2 performance among carbapenem-non-susceptible *P. aeruginosa* met ISO acceptance criteria only for ceftazidime/avibactam (Table 1).

Among all isolates, including *Enterobacterales* and *P. aeruginosa*, VITEK 2 performance met ISO acceptance criteria only for ceftazidime/avibactam and failed for the remaining agents (Table 1).

### Performance of VITEK 2 for colistin

**Fig 3:**
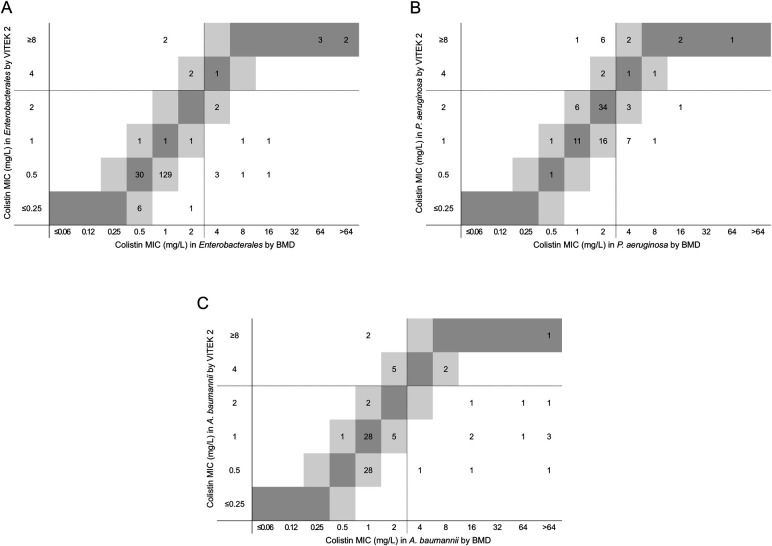
Comparison of colistin MICs between VITEK 2 and BMD in carbapenem-non-susceptible gram-negative isolates. Colistin MICs for *Enterobacterales* (**A**), *P. aeruginosa* (**B**), and *A. baumannii* (**C**) isolates were compared between the two methods. Dark gray represents identical MICs, while light gray indicates a twofold MIC difference. Dotted lines correspond to CLSI breakpoints for each antimicrobial.

Table 2 presents the performance of VITEK 2 relative to BMD for colistin in carbapenem-non-susceptible gram-negative bacilli. Based on BMD results, colistin resistance rates were 8.5% for *Enterobacterales*, 19.6% for *P. aeruginosa*, and 17.4% for *A. baumannii*. CA and EA for colistin met the ISO acceptance criteria (≥90%) in *Enterobacterales* but fell below 90% in *P. aeruginosa* and *A. baumannii*, failing to meet the criteria (Table 2). Additionally, VITEK 2 tended to underestimate colistin MICs in *Enterobacterales* and *A. baumannii* compared to BMD (Fig. 3). As there is no susceptible category for colistin, VME and ME could not be assessed. For all species combined, VITEK 2 demonstrated a CA of 85.4% and an EA of 89.2% for colistin, failing to meet the ISO acceptance criteria (Table 2). BMD MIC distributions by organism group for novel BL/BLI combinations and colistin are shown in Fig. S1, with corresponding MIC ranges and MIC_50_ and MIC_90_ values provided in Table S3.

**TABLE 2:** Performance of VITEK 2 for colistin in carbapenem-non-susceptible gram-negative isolates^*b*^

Organism	VITEK 2	BMD		No. (%) of errors			CA (%)	EA (%)
		I	R	VME	ME	mE		
*Enterobacterales* (*n* = 188)^*a*^	I	168	10	N/A	N/A	14 (7.4)	92.6	94.7
	R	4	6					
*K*. *pneumoniae*	I	62	7	N/A	N/A	10 (12.8)	87.2	92.3
	R	3	6					
*E*. *coli*	I	52	1	N/A	N/A	2 (3.7)	96.3	96.3
	R	1	0					
*K*. *aerogenes*	I	23	0	N/A	N/A	0 (0)	100	100
	R	0	0					
*C*. *freundii* complex	I	22	0	N/A	N/A	0 (0)	100	100
	R	0	0					
*K*. *oxytoca*	I	6	2	N/A	N/A	2 (25.0)	75.0	75.0
	R	0	0					
*C*. *koseri*	I	3	0	N/A	N/A	0 (0)	100	100
	R	0	0					
*P*. *aeruginosa* (*n* = 97)	I	69	12	N/A	N/A	21 (21.6)	78.4	83.5
	R	9	7					
*A*. *baumannii* (*n* = 86)	I	64	12	N/A	N/A	19 (22.1)	77.9	83.7
	R	7	3					
All species combined (*n* = 371)	I	301	34	N/A	N/A	54 (14.6)	85.4	89.2
	R	20	16					

^*a*^ *S. marcescens* and *P. mirabilis* isolates were excluded due to intrinsic resistance to colistin, while *E. cloacae* complex isolates were excluded because the VITEK 2 AST-N439 card does not report colistin susceptibility results.

^*b*^ BMD, broth microdilution; CA, categorical agreement; EA, essential agreement; I, intermediate; ME, major error; mE, minor error; N/A, not applicable; R, resistant; VME, very major error.

## DISCUSSION

Novel BL/BLI combinations have emerged as important treatment options for managing MDR gram-negative bacterial infections; however, delays in their inclusion in commercial AST panels impede their effective use in clinical practice (20). Additionally, accurate colistin susceptibility testing is essential for improving patient outcomes and limiting the spread of resistance. Yet, commercial AST panels for colistin have shown unreliable performance, making routine AST for colistin in clinical microbiology laboratories challenging to implement (21–23). This study is the first to evaluate the performance of the VITEK 2 AST-N439 card, a long-awaited commercial AST panel that includes novel BL/BLI combinations—ceftazidime/avibactam, ceftolozane/tazobactam, imipenem/relebactam, and meropenem/vaborbactam—along with a new formulation of colistin (cs02n). Our findings revealed that the VITEK 2 AST-N439 card has suboptimal performance for the novel BL/BLI combinations and remains unreliable in detecting colistin resistance, even with the new formulation, in carbapenem-non-susceptible gram-negative isolates, the primary target group for this card.

The VITEK 2 AST-N439 card package insert claims that performance for novel BL/BLI combinations in *Enterobacterales* and *P. aeruginosa* meets ISO acceptance criteria (24); however, our findings did not support this, except for ceftazidime/avibactam in *P. aeruginosa*. These results also contrast with previous studies, which reported excellent performance of VITEK 2 for novel BL/BLI combinations in *Enterobacterales* and *P. aeruginosa*. Unlike our study, which included a sufficient number of carbapenem-non-susceptible isolates, those studies evaluated only a limited number of challenge isolates, such as CRE and CRPA isolates (12–14). This difference in isolate selection may explain the observed discrepancies in VITEK 2 performance. Other studies that included only challenge isolates reported suboptimal VITEK 2 performance for novel BL/BLI combinations (15, 16), which aligns with our findings.

In Korea, ceftazidime/avibactam and ceftolozane/tazobactam are the only approved novel BL/BLI combinations, approved in December 2022 and April 2017, respectively. However, these drugs were infrequently prescribed due to their high cost until they were included in the national health insurance reimbursement list—ceftolozane/tazobactam in October 2022 and ceftazidime/avibactam in February 2024. In addition, the global supply of ceftolozane/tazobactam was temporarily interrupted from December 2020 to January 2022, further limiting its use (25). Since reimbursement became available and the supply issue was resolved, the use of ceftazidime/avibactam and ceftolozane/tazobactam has increased significantly; consequently, resistance is expected to rise. In our study, the resistance rate to ceftolozane/tazobactam in carbapenem-non-susceptible *Pseudomonas aeruginosa* increased after the introduction of reimbursement in October 2022 (before: 25.6% [11 out of 43]; after: 37.0% [20 out of 54]). Given this trend, ongoing surveillance and antimicrobial stewardship are essential to preserve the efficacy of ceftolozane/tazobactam. In our study, the resistance rate to ceftazidime/avibactam was low (8.3%) in carbapenem-non-susceptible *Enterobacterales*. The majority of ceftazidime/avibactam-resistant isolates (75% [15 out of 20]) were metallo-β-lactamase (MBL)-producing *Enterobacterales*, which exhibited cross-resistance to imipenem/relebactam and meropenem/vaborbactam. MBL-producing *Enterobacterales* are known to be resistant to ceftazidime/avibactam, imipenem/relebactam, and meropenem/vaborbactam, leaving very limited treatment options for infections caused by these organisms (4, 26). Alarmingly, the widespread use of ceftazidime/avibactam has led to MBLs surpassing KPC as the predominant carbapenemase in some hospitals (27). In Korea, the recent expansion of ceftazidime/avibactam availability highlights the pressing need for active surveillance of MBL-producing *Enterobacterales* and effective antimicrobial stewardship to preserve its efficacy.

Currently, both the CLSI and the European Committee on Antimicrobial Susceptibility Testing (EUCAST) recommend BMD, in accordance with ISO 20776-1, as the sole valid method for colistin susceptibility testing (28). However, because it is labor intensive and time-consuming, most clinical microbiology laboratories do not routinely perform this reference method. Consequently, disk diffusion, gradient diffusion, and automated systems such as VITEK 2 remain widely used for colistin susceptibility testing, despite CLSI and EUCAST advising against their use (21, 22). VITEK 2 had previously been reported to show high agreement with BMD for colistin susceptibility testing (29, 30). However, more recent studies have shown that VITEK 2 failed to identify colistin resistance in a significant proportion of colistin-resistant gram-negative isolates (21–23, 31, 32). In May 2022, bioMérieux announced a reformulation of colistin, claiming that the new cs02n formulation demonstrated improved performance compared to the previous cs01n formulation. The cs02n formulation was incorporated into the newly introduced VITEK 2 AST-N439 card. According to the package insert released in August 2022, CA and EA for colistin were reported as 94.2% and 92.3%, respectively, in gram-negative isolates, meeting the ISO acceptance criteria (24). In contrast, our study found CA and EA for colistin to be 85.4% and 89.2%, respectively, in gram-negative isolates, which failed to meet these criteria. Notably, the AST-N439 card detected colistin resistance in only 32.0% of colistin-resistant gram-negative isolates. As the manufacturer has not disclosed details of the colistin reformulation or specific performance data, it remains unclear why this reformulation performed worse than expected. Nevertheless, our findings highlight that, despite the colistin reformulation, VITEK 2 remains an unreliable method for colistin susceptibility testing.

A major limitation of this study is the use of colonies from separate plates to prepare inocula for VITEK 2 and BMD testing, which may have contributed to discrepancies between the two methods. Although triplicate BMD testing with separate bacterial suspensions was performed to resolve VMEs and MEs, conducting triplicate testing with both VITEK 2 and BMD would have been more appropriate for addressing these discrepancies. In addition, we did not evaluate the performance of VITEK 2 according to resistance mechanism type. Given that the efficacy of novel BL/BLI combinations can vary, depending on the type of extended-spectrum β-lactamase or carbapenemase (3, 5, 33, 34), further studies are necessary to evaluate VITEK 2 performance in isolates with specific resistance mechanisms. Another limitation is the small number of resistant isolates for novel BL/BLI combinations included in our study. As a result, one or two VMEs led to failure in meeting the ISO criterion of ≤3%. According to ISO recommendations, when a significant number of resistant isolates are not included, a VME rate of >3% may be acceptable (19). Additional studies should incorporate more resistant isolates to accurately assess the VME rate. The criteria used to define carbapenem non-susceptibility in this study may also represent a limitation. A considerable proportion of isolates classified as intermediate or resistant to at least one carbapenem by VITEK 2 may, in fact, be carbapenem susceptible. As a result, the performance of VITEK 2 in these isolates may not accurately reflect its performance in truly carbapenem-resistant isolates. Nonetheless, automated systems such as VITEK 2 are routinely used for AST in clinical laboratories. Accordingly, carbapenem-non-susceptible isolates identified by VITEK 2 are typically considered candidates for testing with the AST-N439 card, as is done in our laboratory. Therefore, we believe the findings of our study remain applicable to routine clinical practice. Lastly, we did not assess the other agents (amikacin, amoxicillin/clavulanic acid, cefepime, ceftazidime, ciprofloxacin, fosfomycin, gentamicin, meropenem, piperacillin/tazobactam, and tigecycline) included in the VITEK 2 AST-N439 card, other than the novel BL/BLI combinations and colistin.

In conclusion, our study highlights the suboptimal performance of VITEK 2 for novel BL/BLI combinations in carbapenem-non-susceptible gram-negative isolates. Furthermore, despite modifications to the colistin formulation in the VITEK 2 card, it remains an unreliable method for detecting colistin resistance.

## SUPPLEMENTAL MATERIAL

Figure S1 spectrum.00166-25-s0001.tif BMD MIC distributions for novel BL/BLI combinations and colistin, shown by organism group.

Supplemental tables spectrum.00166-25-s0002.docx Tables S1 to S3

ASM does not own the copyrights to Supplemental Material that may be linked to, or accessed through, an article. The authors have granted ASM a non-exclusive, world-wide license to publish the Supplemental Material files. Please contact the corresponding author directly for reuse.
