# Supplementary figures and images for: Evaluation of the VITEK 2 AST-N439 card for susceptibility testing of novel β-lactam/β-lactamase inhibitor combinations and colistin in carbapenem-non-susceptible gram-negative bacilli
Source: Microbiol Spectr. 2025 Aug 21;13(10):e00166-25. doi: 10.1128/spectrum.00166-25 (PMC12502683; doi:10.1128/spectrum.00166-25)

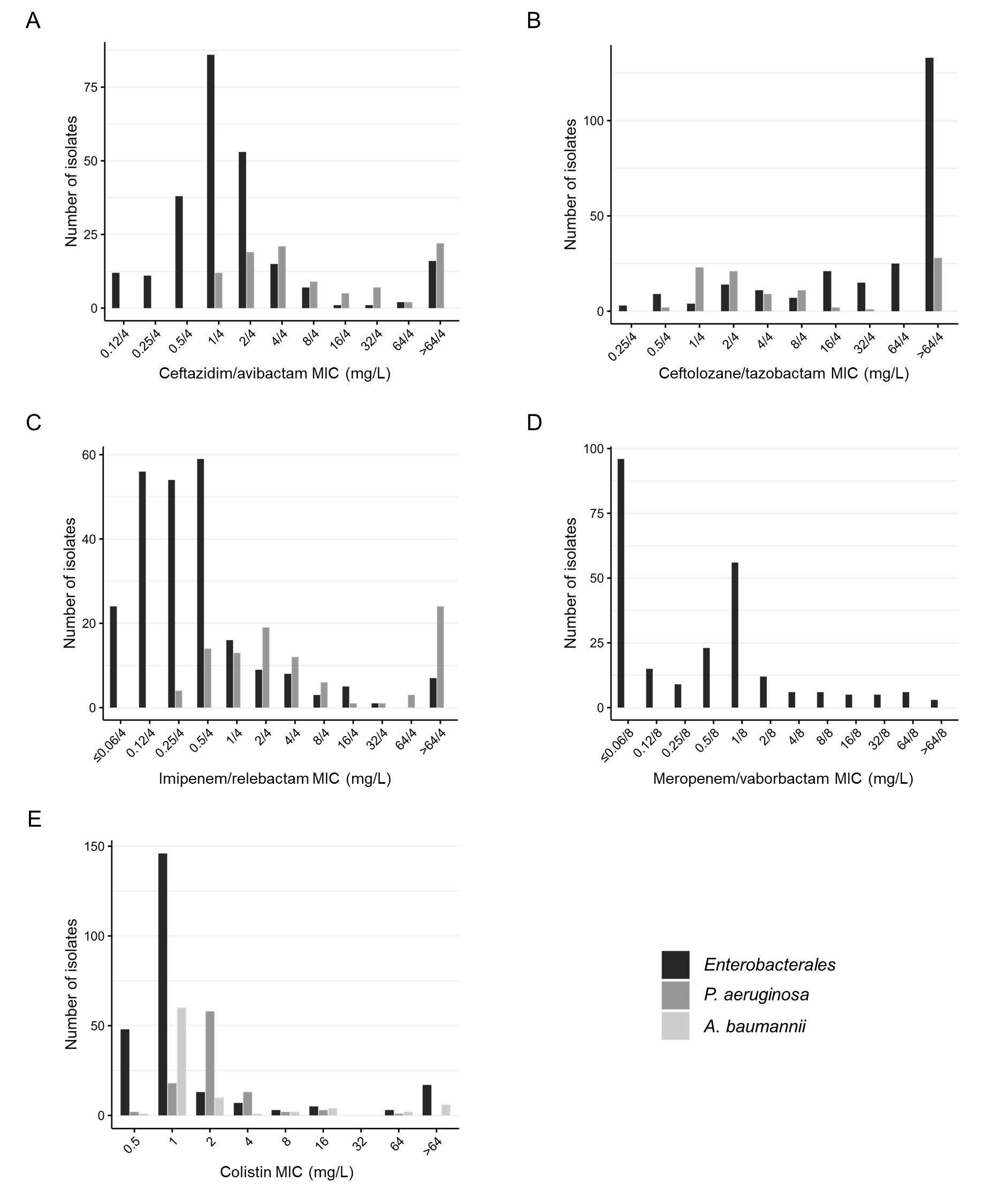

Supplement: Figure S1 — BMD MIC distributions for novel BL/BLI combinations and colistin, shown by organism group. [file spectrum.00166-25-s0001.tif]
