# Supplementary material for: Evaluation of the VITEK 2 AST-N439 card for susceptibility testing of novel β-lactam/β-lactamase inhibitor combinations and colistin in carbapenem-non-susceptible gram-negative bacilli
Source: Microbiol Spectr. 2025 Aug 21;13(10):e00166-25. doi: 10.1128/spectrum.00166-25 (PMC12502683; doi:10.1128/spectrum.00166-25)
Supplement: Supplemental tables — Tables S1 to S3 [file spectrum.00166-25-s0002.docx]

Table S1. Carbapenemase profiles of carbapenem-non-susceptible isolates included in this study

| Carbapenemase type | *K*. *pneumoniae* | *E.* *coli* | *E.* *cloacae* complex | *K. aerogenes* | *C. freundii* complex | *S. marcescens* | *K. oxytoca* | *C. koseri* | *P.* *mirabilis* | *P. aeruginosa* | *A. baumannii* |
| --- | --- | --- | --- | --- | --- | --- | --- | --- | --- | --- | --- |
| KPC | 38 | 15 |  |  | 5 | 1 | 1 | 2 |  |  |  |
| GES |  |  |  |  |  |  |  |  |  | 2 |  |
| NDM | 6 | 4 | 1 |  |  |  | 1 |  | 1 | 14 |  |
| VIM |  |  |  |  |  |  | 1 |  |  | 6 |  |
| IMP |  |  |  |  |  |  |  |  |  | 2 |  |
| OXA-23 |  |  |  |  |  |  |  |  |  |  | 86 |
| OXA-48 |  | 2 | 1 |  |  |  |  | 1 |  |  |  |
| NDM + GES |  |  |  |  |  |  |  |  |  | 1 |  |
| NDM + OXA-48-like |  | 1 |  |  |  |  |  |  |  |  |  |
| Not detected | 34 | 32 | 36 | 23 | 17 | 12 | 5 |  | 2 | 72 |  |
| Total | 78 | 54 | 38 | 23 | 22 | 13 | 8 | 3 | 3 | 97 | 86 |

Table S2. Performance of VITEK 2 for novel BL/BLI combinations in carbapenem-non-susceptible *Enterobacterales*, detailed by species

| Organism/antimicrobial agent | Total tested | BMD | | |  | No. (%) of errors | | |  | CA, % | EA, % |
| --- | --- | --- | --- | --- | --- | --- | --- | --- | --- | --- | --- |
|  |  | S | I | R |  | VME | ME | mE |  |  |  |
| *K*. *pneumoniae* |  |  |  |  |  |  |  |  |  |  |  |
| Ceftazidime/avibactam | 78 | 68 | N/A | 10 |  | 0 (0) | 6 (8.8) | N/A |  | 92.3 | 89.7 |
| Ceftolozane/tazobactam | 77^*^ | 2 | 0 | 75 |  | 3 (4.0) | 0 (0) | 0 (0) |  | 96.1 | 89.6 |
| Imipenem/relebactam | 78 | 66 | 4 | 8 |  | 0 (0) | 0 (0) | 6 (7.7) |  | 92.3 | 98.7 |
| Meropenem/vaborbactam | 78 | 68 | 4 | 6 |  | 0 (0) | 5 (7.4) | 3 (3.8) |  | 89.7 | 93.6 |
| *E*. *coli* |  |  |  |  |  |  |  |  |  |  |  |
| Ceftazidime/avibactam | 54 | 49 | N/A | 5 |  | 1 (20.0) | 0 (0) | N/A |  | 98.1 | 90.7 |
| Ceftolozane/tazobactam | 53^*^ | 2 | 4 | 47 |  | 7 (14.9) | 0 (0) | 2 (3.8) |  | 83.0 | 79.2 |
| Imipenem/relebactam | 54 | 46 | 1 | 7 |  | 1 (14.3) | 0 (0) | 1 (1.9) |  | 96.3 | 85.7 |
| Meropenem/vaborbactam | 54 | 47 | 1 | 6 |  | 1 (16.7) | 0 (0) | 1 (1.9) |  | 96.3 | 98.1 |
| *E*. *cloacae* complex |  |  |  |  |  |  |  |  |  |  |  |
| Ceftazidime/avibactam | 38 | 37 | N/A | 1 |  | 0 (0) | 0 (0) | N/A |  | 100 | 81.6 |
| Ceftolozane/tazobactam | 37^*^ | 7 | 2 | 28 |  | 5 (17.9) | 2 (28.6) | 3 (8.1) |  | 73.0 | 51.4 |
| Imipenem/relebactam | 38 | 35 | 1 | 2 |  | 0 (0) | 1 (2.9) | 1 (2.6) |  | 94.7 | 97.4 |
| Meropenem/vaborbactam | 38 | 37 | 0 | 1 |  | 0 (0) | 0 (0) | 1 (2.6) |  | 97.4 | 97.4 |
| *K*. *aerogenes* |  |  |  |  |  |  |  |  |  |  |  |
| Ceftazidime/avibactam | 23 | 23 | N/A | 0 |  | 0 (N/A) | 0 (0) | N/A |  | 100 | 95.7 |
| Ceftolozane/tazobactam | 23 | 8 | 1 | 14 |  | 0 (0) | 0 (0) | 1 (4.3) |  | 95.7 | 73.9 |
| Imipenem/relebactam | 23 | 23 | 0 | 0 |  | 0 (N/A) | 0 (0) | 0 (0) |  | 100 | 100 |
| Meropenem/vaborbactam | 23 | 23 | 0 | 0 |  | 0 (N/A) | 1 (4.3) | 0 (0) |  | 95.7 | 95.7 |
| *C*. *freundii* complex |  |  |  |  |  |  |  |  |  |  |  |
| Ceftazidime/avibactam | 22 | 22 | N/A | 0 |  | 0 (N/A) | 0 (0) | N/A |  | 100 | 86.4 |
| Ceftolozane/tazobactam | 21^*^ | 2 | 0 | 19 |  | 0 (0) | 0 (0) | 0 (0) |  | 100 | 66.7 |
| Imipenem/relebactam | 22 | 21 | 1 | 0 |  | 0 (N/A) | 0 (0) | 0 (0) |  | 100 | 95.5 |
| Meropenem/vaborbactam | 22 | 22 | 0 | 0 |  | 0 (N/A) | 0 (0) | 0 (0) |  | 100 | 100 |
| *S*. *marcescens* |  |  |  |  |  |  |  |  |  |  |  |
| Ceftazidime/avibactam | 13 | 13 | N/A | 0 |  | 0 (N/A) | 0 (0) | N/A |  | 100 | 69.2 |
| Ceftolozane/tazobactam | 13 | 7 | 2 | 4 |  | 0 (0) | 0 (0) | 2 (15.4) |  | 84.6 | 76.9 |
| Imipenem/relebactam | 13 | 10 | 2 | 1 |  | 0 (0) | 0 (0) | 2 (15.4) |  | 84.6 | 92.3 |
| Meropenem/vaborbactam | 13 | 11 | 0 | 2 |  | 0 (0) | 1 (9.1) | 0 (0) |  | 92.3 | 84.6 |
| *K*. oxytoca |  |  |  |  |  |  |  |  |  |  |  |
| Ceftazidime/avibactam | 8 | 6 | N/A | 2 |  | 0 (0) | 0 (0) | N/A |  | 100 | 75.0 |
| Ceftolozane/tazobactam | 8 | 2 | 2 | 4 |  | 0 (0) | 0 (0) | 1 (12.5) |  | 87.5 | 100 |
| Imipenem/relebactam | 8 | 6 | 0 | 2 |  | 0 (0) | 0 (0) | 0 (0) |  | 100 | 100 |
| Meropenem/vaborbactam | 8 | 7 | 0 | 1 |  | 1 (100) | 0 (0) | 1 (12.5) |  | 75.0 | 87.5 |
| *C*. *koseri* |  |  |  |  |  |  |  |  |  |  |  |
| Ceftazidime/avibactam | 3 | 3 | N/A | 0 |  | 0 (N/A) | 0 (0) | N/A |  | 100 | 100 |
| Ceftolozane/tazobactam | 3 | 0 | 0 | 3 |  | 0 (0) | 0 (N/A) | 0 (0) |  | 100 | 66.7 |
| Imipenem/relebactam | 3 | 2 | 0 | 1 |  | 0 (0) | 0 (0) | 1 (33.3) |  | 66.7 | 100 |
| Meropenem/vaborbactam | 3 | 2 | 1 | 0 |  | 0 (N/A) | 0 (0) | 1 (33.3) |  | 66.7 | 66.7 |
| *P*. *mirabilis* |  |  |  |  |  |  |  |  |  |  |  |
| Ceftazidime/avibactam | 3 | 1 | N/A | 2 |  | 0 (0) | 0 (0) | N/A |  | 100 | 66.7 |
| Ceftolozane/tazobactam | 3 | 0 | 0 | 3 |  | 0 (0) | 0 (N/A) | 0 (0) |  | 100 | 66.7 |
| Imipenem/relebactam | 3 | 0 | 0 | 3 |  | 0 (0) | 0 (N/A) | 0 (0) |  | 100 | 100 |
| Meropenem/vaborbactam | 3 | 0 | 0 | 3 |  | 1 (33.3) | 0 (N/A) | 0 (0) |  | 66.7 | 66.7 |
| All species combined |  |  |  |  |  |  |  |  |  |  |  |
| Ceftazidime/avibactam | 242 | 222 | N/A | 20 |  | 1 (5.0) | 6 (2.7) | N/A |  | 97.1 | 87.2 |
| Ceftolozane/tazobactam | 238 | 30 | 11 | 197 |  | 15 (7.6) | 2 (6.7) | 9 (3.8) |  | 89.1 | 76.9 |
| Imipenem/relebactam | 242 | 209 | 9 | 24 |  | 1 (4.2) | 1 (0.5) | 11 (4.5) |  | 94.6 | 97.9 |
| Meropenem/vaborbactam | 242 | 217 | 6 | 19 |  | 3 (15.8) | 7 (3.2) | 7 (2.9) |  | 93.0 | 94.6 |

^*^Four isolates (one *K*. *pneumoniae*, one *E*. *coli*, one *E*. *cloacae* complex, and one *C*. *freundii* complex) were excluded from the analysis because ceftolozane/tazobactam MIC testing on the VITEK 2 system was terminated due to insufficient growth in the positive control well.

Abbreviations: BMD, broth microdilution; S, susceptible; I, intermediate; R, resistant; VME, very major error; ME, major error; mE, minor error; CA, categorical agreement; EA, essential agreement; N/A, not applicable.

Table S3. BMD MIC ranges, MIC_50_ and MIC_90_ values for novel BL/BLI combinations and colistin, presented by organism group

| Organism/antimicrobial agent | MIC range (mg/L) | MIC_50_ (mg/L) | MIC_90_ (mg/L) |
| --- | --- | --- | --- |
| *Enterobacterales* |  |  |  |
| Ceftazidime/avibactam | 0.12/4–>64/4 | 1/4 | 8/4 |
| Ceftolozane/tazobactam | 0.25/4–>64/4 | >64/4 | >64/4 |
| Imipenem/relebactam | ≤0.06/4–>64/4 | 0.25/4 | 2/4 |
| Meropenem/vaborbactam | ≤0.06/8–>64/8 | 0.5/8 | 8/8 |
| Colistin | 0.5–>64 | 1 | 16 |
| *P*. *aeruginosa* |  |  |  |
| Ceftazidime/avibactam | 1/4–>64/4 | 4/4 | >64/4 |
| Ceftolozane/tazobactam | 0.5/4–>64/4 | 4/4 | >64/4 |
| Imipenem/relebactam | 0.25/4–>64/4 | 2/4 | >64/4 |
| Colistin | 0.5–64 | 2 | 4 |
| *A*. *baumannii* |  |  |  |
| Colistin | 0.5–>64 | 1 | 16 |
| All species combined |  |  |  |
| Ceftazidime/avibactam | 0.12/4–>64/4 | 2/4 | >64/4 |
| Ceftolozane/tazobactam | 0.25/4–>64/4 | 64/4 | >64/4 |
| Imipenem/relebactam | ≤0.06/4–>64/4 | 0.5/4 | 64/4 |
| Meropenem/vaborbactam | ≤0.06/8–>64/8 | 0.5/8 | 8/8 |
| Colistin | 0.5–>64 | 1 | 8 |

Abbreviation: MIC, minimum inhibitory concentration.

Fig. S1. BMD MIC distributions for novel BL/BLI combinations and colistin, shown by organism group
